# Adjuvant use of topical metformin with standard therapies in recalcitrant central centrifugal cicatricial alopecia: A case series

**DOI:** 10.1016/j.jdcr.2024.12.025

**Published:** 2025-01-04

**Authors:** Monica N. Williams, Janeth R. Campbell, Sach Thakker, Monique Chheda

**Affiliations:** a Department of Dermatology, MedStar Georgetown University Hospital/MedStar Washington Hospital Center, Washington, District of Columbia; b Georgetown University School of Medicine, Washington, District of Columbia

**Keywords:** alopecia, central centrifugal cicatricial alopecia, clinical features, metabolic dysfunction, metformin, treatment strategies

## Introduction

Central centrifugal cicatricial alopecia (CCCA) is an inflammatory, scarring alopecia that usually presents with characteristic patches of hair loss beginning on the vertex scalp with centrifugal spread. It occurs most often in middle-aged Black women and is commonly associated with burning, dysesthesia, tenderness, or pruritus; though it may be asymptomatic. Perifollicular hyperpigmentation may also be seen on dermoscopy.^1^ Data are limited regarding the treatment of CCCA, with most therapeutic strategies targeting hair regrowth and prevention of disease progression. Topical minoxidil, topical steroids, intralesional steroids, antifungal shampoo, and oral tetracyclines have all been used to treat CCCA. Nevertheless, CCCA remains a difficult condition to treat, with patients often experiencing significant negative impact on their quality of life.^2^ Recent studies have demonstrated the utility of topical metformin for the treatment of CCCA.^3^ Here, we report 3 additional cases of recalcitrant CCCA treated with topical metformin in addition to standard therapy.

## Case 1

**Fig 1:**
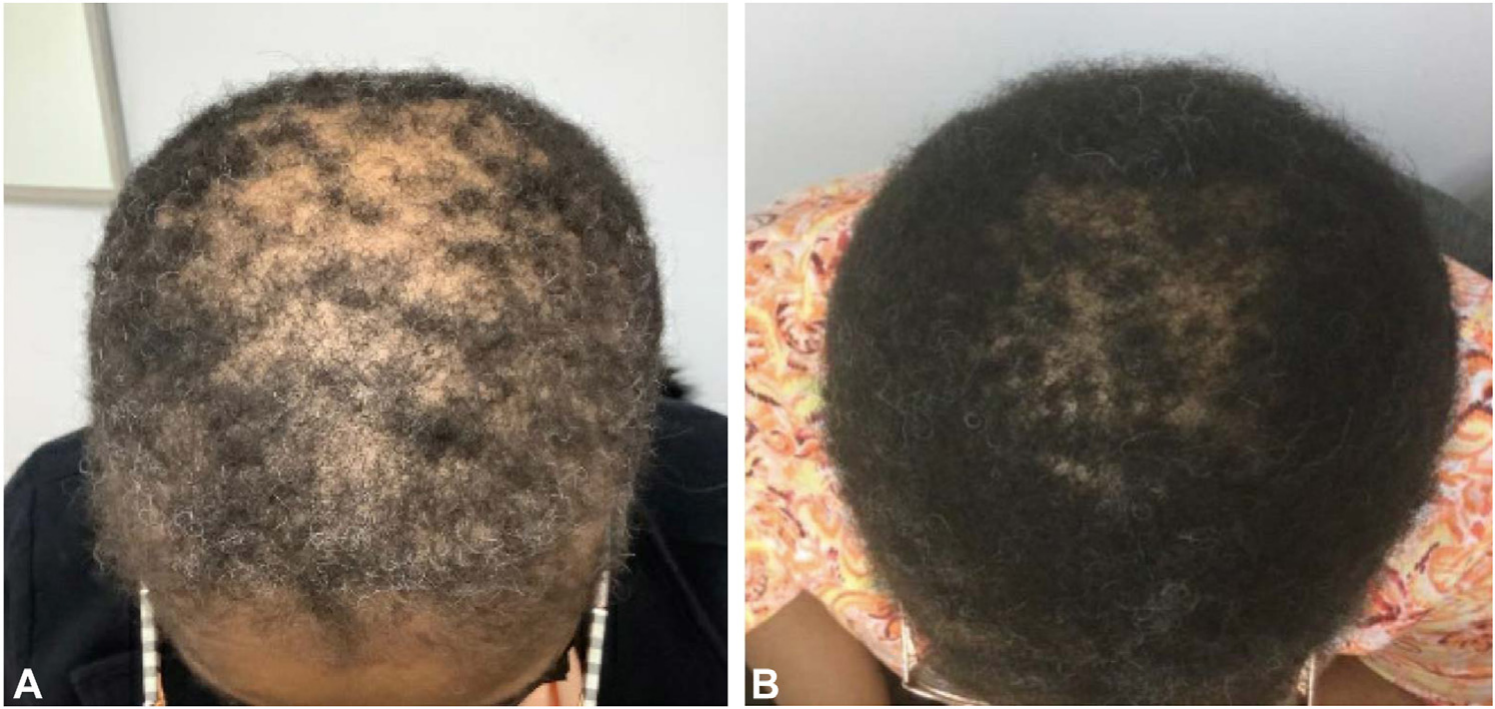
Improvement of CCCA after application of topical metformin in case 1. **A,** CCCA in patient 1 before initiation of topical metformin. **B,** Improvement in hair density after 6 m of topical minoxidil 5% compounded with 10% metformin treatment nightly. CCCA, central centrifugal cicatricial alopecia.

Patient 1 is a 51-year-old Black female who presented with a 3-year history of alopecia, which was subsequently diagnosed as CCCA via histopathologic examination. The patient was previously treated with doxycycline 100 mg daily, fluocinonide 0.05% solution, clobetasol 0.05% ointment, ketoconazole shampoo, and minoxidil 5% compounded with triamcinolone 0.1% solution with only mild improvement. Significant improvement in hair density was observed 6 months after the addition of nightly compounded minoxidil 5% with 10% metformin cream. (Fig 1).

## Case 2

**Fig 2:**
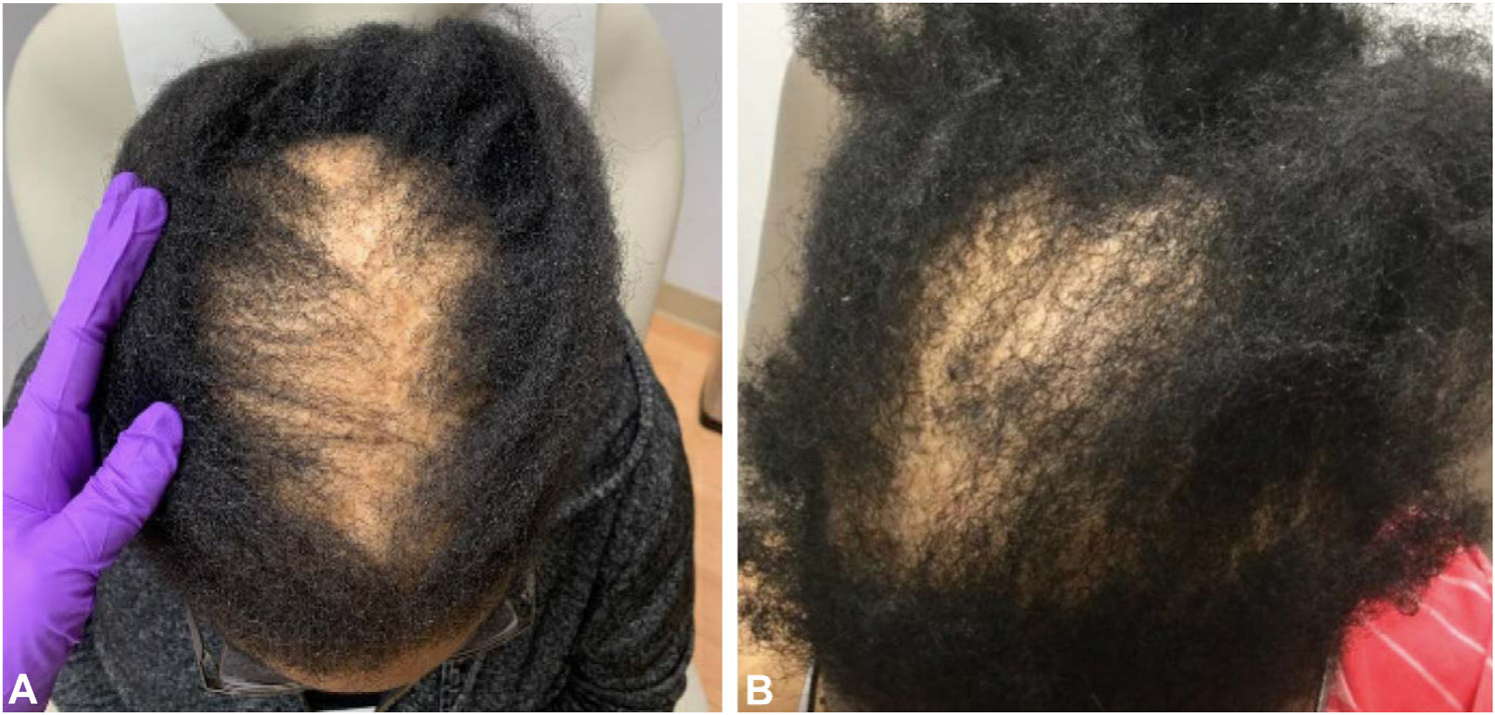
Improvement of CCCA after application of topical metformin in case 2. **A,** CCCA in patient 2 before initiation of topical metformin. **B,** Improvement in hair density and regrowth after 10 m of topical metformin 10% cream nightly, minoxidil 6% compounded with triamcinolone 0.1% daily, and monthly intralesional triamcinolone injections 10 mg/ml. CCCA, central centrifugal cicatricial alopecia.

Patient 2 is a 32-year-old Black female with a past medical history of diabetes mellitus, hidradenitis suppurativa, and polycystic ovarian syndrome with CCCA confirmed via histopathologic examination. She was previously treated with topical minoxidil 6% compounded with triamcinolone 0.1%, intralesional triamcinolone injections, oral doxycycline 100 mg daily, and compounded minoxidil 6% and tacrolimus 0.1% solution with minimal improvement. She was therefore started on topical metformin 10% cream nightly. She also continued use of minoxidil compounded with tacrolimus solution daily and monthly intralesional triamcinolone injections 10 mg/ml. At 10 months follow up, there was notable improvement in hair density, and hair regrowth was observed (Fig 2).

## Case 3

**Fig 3:**
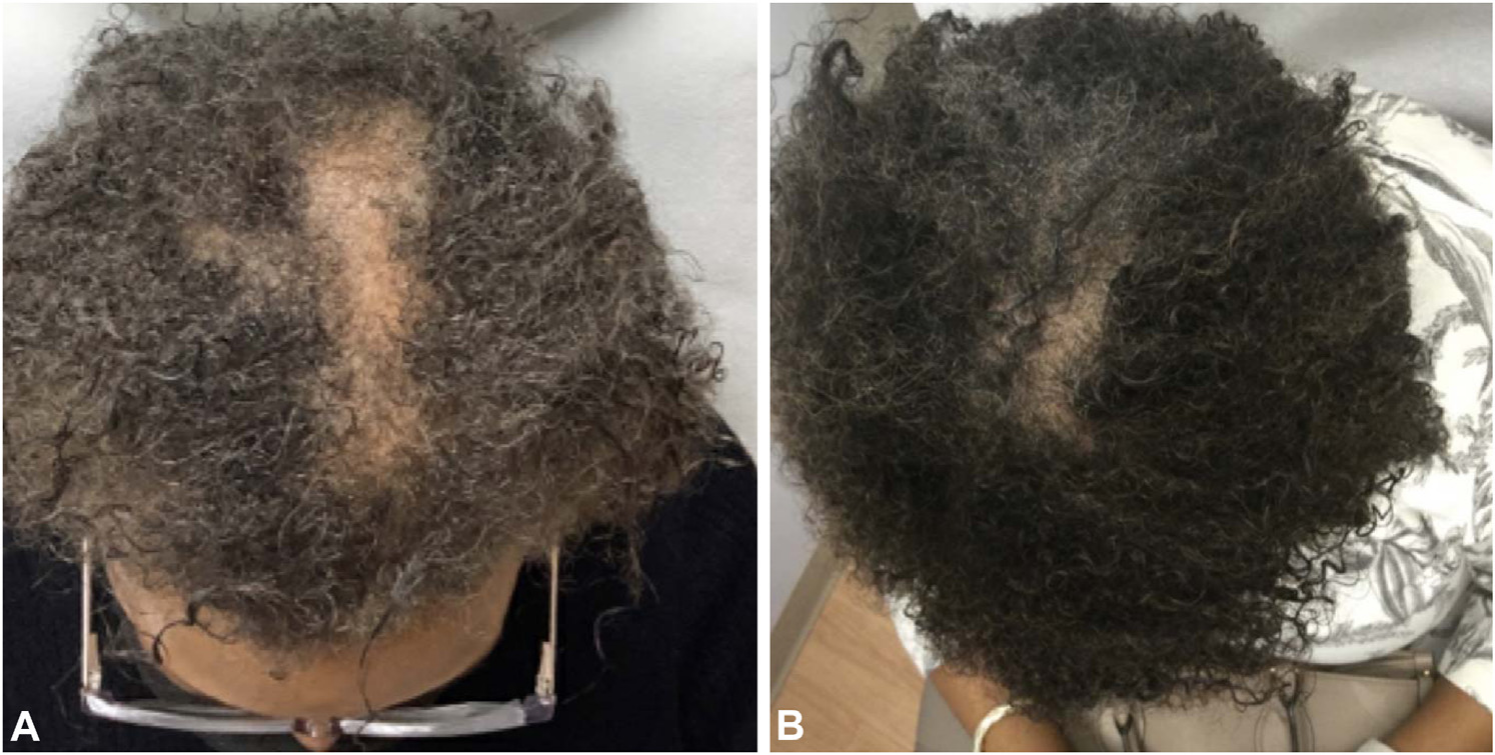
Improvement of CCCA after application of topical metformin in case 3. **A,** CCCA in patient 3 before initiation of topical metformin. **B,** Improvement in hair density and regrowth after 10 m of topical metformin 10% compound daily, doxycycline 100 mg daily for 6 weeks, and topical minoxidil 7% compounded with triamcinolone daily.

Patient 3 is a 62-year-old Black female with CCCA confirmed via histopathologic examination, previously treated with topical minoxidil 5% foam daily and 0.05% fluocinonide solution three times weekly with little reported improvement. She subsequently started a 6-week course of doxycycline 100 mg daily, topical minoxidil 7% compounded with triamcinolone 0.1% solution daily and compounded metformin 10% daily with significant improvement in hair density and regrowth at 10 months follow-up. (Fig 3).

## Discussion

Treatment of CCCA aims to encourage hair regrowth and prevent disease progression. While there are no clear guidelines for management, therapy is multifaceted and may involve inflammatory, hormonal, and mechanical processes. Maintenance therapy for CCCA often includes a mid-potency topical steroid several times per week, topical minoxidil daily, an anti-dandruff shampoo every 1-2 weeks, and intralesional steroids as needed.^4^ Minimal hair grooming and avoiding excessive traction of hairs is also recommended.^5^ Anti-inflammatory treatment of CCCA includes weekly anti-dandruff shampoo, daily high potency topical steroids, intralesional steroids, and oral tetracyclines.^6^ Minoxidil in concentrations of 2% to 5% has been used topically to treat various types of alopecia. A vasodilator, minoxidil is hypothesized to work via acceleration of growth factor secretions, and has been shown to increase the proportion of terminal to vellus hairs by prolonging the anagen phase hair cycle at concentrations of 5%.^6^^,^^7^

Metformin is a first line treatment for the management of diabetes mellitus type 2 and works via the inhibition of hepatic gluconeogenesis.^8^ Most recently, Araoye et al also observed substantial regrowth in 2 histopathologically confirmed cases of CCCA patients with use of topical 10% metformin cream alone. They proposed metformin's reduction of circulating androgens along and anti-fibrotic effects as a potential mechanism of action. Prominent fibrosis is a notable feature of CCCA’s pathogenesis.^3^^,^^9^ Furthermore, patients with CCCA demonstrate an increased incidence of disorders of fibroproliferation, such as uterine fibroids, supporting a fibroproliferative dysregulatory process.^10^ Notably, metformin has also demonstrated efficacy in the treatment of idiopathic pulmonary fibrosis in mouse models via modulation of the Insulin-like Growth Factor 1.^11^

Central scalp alopecia is associated with endocrine disorders, namely diabetes mellitus type 2, which has been independently associated with an increased risk of severe central scalp hair loss in African American women.^12^ Obese women with CCCA also have a 4 times higher odds of having diabetes mellitus type 2 when compared to race, age, and sex-matched controls, suggesting the role of metabolic dysfunction in its pathogenesis.^13^ Notably, a retrospective review of 100 CCCA patients found that individuals who were taking oral metformin were significantly more likely to improve after treatment.^14^ Metformin acts as an activator of the AMPK enzyme, which is encoded by the PRKAA2 gene. Interestingly, this gene has been revealed to be underexpressed in one-third of CCCA patients.^15^ While the exact pathophysiology of CCCA remains unknown, the improvement of CCCA with the use of metformin further suggests a common biochemical pathway.^14^^,^^15^

Here, we demonstrate improvement in hair regrowth with the addition of metformin in CCCA patients using standard treatments. Our case series is limited, as we did not prescribe topical metformin as monotherapy to patients with CCCA; multifactorial hair loss was seen in addition to CCCA to include androgenic and traction. Our cases, however, demonstrate improvement in alopecia of varying etiology and distribution with the addition of topical metformin to standard therapies — suggesting its use as a conjunction treatment. Given that these patients benefited from topical metformin after failing standard therapies, the authors recommend considering topical metformin as part of the initial therapy for CCCA. However, large clinical trials examining the use of topical metformin in CCCA, and other types of alopecia, are still warranted.

## Conflicts of interest

None disclosed.
